# Bromido(*meso*-5,5,7,12,12,14-hexa­methyl-1,4,8,11-tetra­aza­cyclo­tetra­deca-1,7-diene)copper(II) bromide dihydrate

**DOI:** 10.1107/S1600536811012852

**Published:** 2011-04-16

**Authors:** Fei-Fei Shi, Xiu-Li He

**Affiliations:** a Ordered Matter Science Research Center, College of Chemistry and Chemical Engineering, Southeast University, Nanjing 210096, People’s Republic of China

## Abstract

There are two formula units (*Z*′ = 2) in the asymmmetric unit of the title compound, [CuBr(C_16_H_32_N_4_)]Br·2H_2_O. The title crystal consists of two [Cu(C_16_H_32_N_4_)]^2+^ cations, two Br^−^ anions and four uncoordinated water mol­ecules. The metal atom is five-coordinate square pyramidal, with a long apical Cu—Br bond [2.9734 (11) and 2.9229 (11) Å in the two cations]. The two cations form a loosely associated dimer through the formation of hydrogen bonds between both N—H and O—H and Br^−^. In addition, there is a network of N—H⋯Br, O—H⋯Br and N—H⋯O hydrogen bonds, leading to the formation of a chain structure.

## Related literature

For the structure of the ligand, see: Maurya *et al.* (1991 ▶); Spirlet *et al.* (1991 ▶). For related macrocyclic complexes, see: Szalda *et al.* (1989 ▶); Tebbe *et al.* (1985 ▶); Whimp *et al.* (1970 ▶) For a description of the geometry of complexes with five-coord­n­ate metal atoms, see: Addison *et al.* (1984 ▶). 
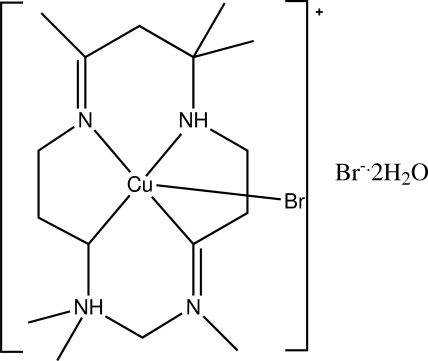


## Experimental

### 

#### Crystal data

[CuBr(C_16_H_32_N_4_)]Br·2H_2_O
*M* *_r_* = 539.85
Monoclinic, 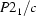

*a* = 17.8747 (16) Å
*b* = 15.5118 (13) Å
*c* = 17.2528 (19) Å
β = 112.073 (1)°
*V* = 4433.0 (7) Å^3^
*Z* = 8
Mo *K*α radiation
μ = 4.61 mm^−1^
*T* = 298 K
0.47 × 0.42 × 0.32 mm

#### Data collection

Rigaku SCXmini diffractometer
Absorption correction: multi-scan (*CrystalClear*; Rigaku, 2005 ▶) *T*_min_ = 0.221, *T*_max_ = 0.320
7814 measured reflections
7814 independent reflections
3668 reflections with *I* > 2σ(*I*)
*R*_int_ = 0.1005

#### Refinement

*R*[*F* ^2^ > 2σ(*F* ^2^)] = 0.048
*wR*(*F* ^2^) = 0.099
*S* = 0.87
7814 reflections
487 parameters
12 restraints
H atoms treated by a mixture of independent and constrained refinement
Δρ_max_ = 0.67 e Å^−3^
Δρ_min_ = −0.82 e Å^−3^

### 

Data collection: *CrystalClear* (Rigaku, 2005 ▶); cell refinement: *CrystalClear*; data reduction: *CrystalClear*; program(s) used to solve structure: *SHELXS97* (Sheldrick, 2008 ▶); program(s) used to refine structure: *SHELXL97* (Sheldrick, 2008 ▶); molecular graphics: *SHELXTL* (Sheldrick, 2008 ▶); software used to prepare material for publication: *SHELXTL*.

## Supplementary Material

Crystal structure: contains datablocks I, global. DOI: 10.1107/S1600536811012852/bv2173sup1.cif

Structure factors: contains datablocks I. DOI: 10.1107/S1600536811012852/bv2173Isup2.hkl

Additional supplementary materials:  crystallographic information; 3D view; checkCIF report

## Figures and Tables

**Table 1:** Hydrogen-bond geometry (Å, °)

*D*—H⋯*A*	*D*—H	H⋯*A*	*D*⋯*A*	*D*—H⋯*A*
O1*W*—H1*W*1⋯Br1	0.82 (2)	2.46 (2)	3.274 (5)	173 (8)
O1*W*—H1*W*2⋯Br2	0.83 (2)	2.58 (3)	3.395 (6)	170 (7)
O2*W*—H2*W*1⋯Br2	0.80 (2)	2.61 (8)	3.238 (6)	136 (9)
O2*W*—H2*W*2⋯Br1	0.81 (2)	2.53 (3)	3.328 (7)	168 (10)
O3*W*—H3*W*1⋯Br4	0.81 (2)	2.62 (3)	3.417 (6)	169 (10)
O3*W*—H3*W*2⋯Br3	0.80 (2)	2.61 (4)	3.362 (6)	156 (8)
O4*W*—H4*W*1⋯Br3	0.81 (2)	2.66 (2)	3.461 (6)	170 (8)
O4*W*—H4*W*2⋯Br4	0.81 (2)	2.58 (4)	3.327 (5)	155 (7)
N1—H1⋯O1*W*	0.91	2.44	3.317 (8)	161
N1—H1⋯Br1	0.91	2.99	3.519 (5)	119
N3—H3⋯Br3^i^	0.91	2.57	3.457 (5)	166
N5—H5⋯Br4	0.91	2.53	3.432 (5)	170
N7—H7⋯O2*W*	0.91	2.40	3.257 (9)	158
N7—H7⋯Br2	0.91	3.14	3.612 (5)	115

Symmetry code: (i) 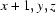.

**Fig. 1.**
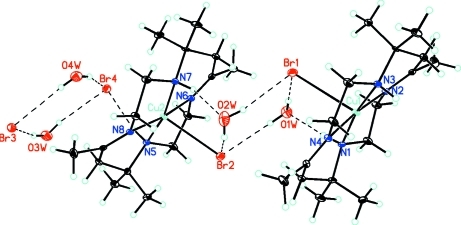
The asymmetric structure of the title molecule, with the atom-numbering scheme. Displacement ellipsoids are drawn at the 30% probability level.

**Fig. 2.**
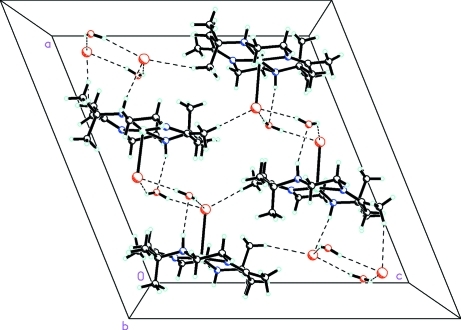
Crystal packing of the compound (I). Hydrogen bonds are shown as dashed lines.

## supplementary crystallographic information

### Comment

The structures of several related macrocyclic complexes have been reported (Whimp *et al.*, 1970; Tebbe *et al.*, 1985). The unsubstituted parent compound exists in the zwitterionic form (Maurya *et al.*, 1991; Spirlet *et al.*, 1991). The copper teraazamacrocyclic complex cation, [Cu(C_16_H_32_N_4_ Br)]+, can combine with different anions to form many kinds of structures.

The title compound, [Cu(C_16_H_32_N_4_Br)]Br^.^2H_2_O, was synthesized by reaction of CuSO_4_^.^5H_2_O and the complex C_18_H_32_N_4_^.^2HBr^.^2H_2_O in methanol solution. The two similar macrocycles are linked *via* N—H···O (water) and O—H···Br hydrogen bonds to form a loosely associated dimer (see Fig. 1). One of the macrocycle units is further associated with the remaining Br anions and water solvent molecules. In the two macrocyclic cations each Cu is square pyramidal five coordinate [τ = 0.07 and 0.11, respectively (Addison *et al.* 1984)] with long Cu—Br bonds [Cu(1)—Br(1), 2.9734 (11); Cu(2)—Br(2), 2.9229 (11)]. The six-membered rings contain double bonds between N(4)—C(4) and N(2)—C(10); N(6)—C(26) and N(8)—C(20) [distances of 1.273 (7) Å and 1.279 (7) Å in molecule 1 and 1.282 (7) and 1.287 (7) Å in molecule 2, respectively]. The average Cu—*N*(amine) and Cu—*N*(imine) bond distances are similar to those found previously (Szalda *et al.*, 1989). The Cu and 4 N atoms are coplanar (r.m.s. average deviation of 0.06 Å). The dihedral "fold" angle between the planes formed by N1, N2, N3 and N1, N4, N3 is 4.39 (2)°. In the crystal structure, the cations, anions and water molecules are linked *via* intermolecular N—H···Br, *O*— H···Br and N—H···O hydrogen bonds forming discrete chains, and the structure is stabilized by intramolecular hydrogen bonds. (see Fig. 2).

### Experimental

All chemicals were of reagent grade and were used as received with out further purification. The precursor complex C_18_H_32_N_4_^.^2HBr^.^2H_2_O was prepared previously. To a 10 ml me thanol solution of CuSO_4_^.^5H_2_O (0.2 mmol,0.087 g), a 5 ml me thanol solution of C_18_H_32_N_4_^.^2HBr^.^2H_2_O (0.2 mmol, 0.0957 g) was added dropwise with stirring. The resulting solution was continuously stirred for about 30 min. Purple crystals suitable for X-ray analysis were obtained by slow evaporation at room temperature over several days.

### Refinement

(type here to add refinement details)

### Crystal data

**Table:**

[CuBr(C_16_H_32_N_4_)]Br·2H_2_O	*F*(000) = 2200
*M**_r_* = 539.85	*D*_x_ = 1.618 Mg m^−^^3^
Monoclinic, *P*2_1_/*c*	Mo *K*α radiation, λ = 0.71073 Å
Hall symbol: -P 2ybc	Cell parameters from 3476 reflections
*a* = 17.8747 (16) Å	θ = 2.3–27.5°
*b* = 15.5118 (13) Å	µ = 4.61 mm^−^^1^
*c* = 17.2528 (19) Å	*T* = 298 K
β = 112.073 (1)°	Prism, green
*V* = 4433.0 (7) Å^3^	0.47 × 0.42 × 0.32 mm
*Z* = 8	

### Data collection

**Table:**

Rigaku SCXmini diffractometer	7814 independent reflections
Radiation source: fine-focus sealed tube	3668 reflections with *I* > 2σ(*I*)
graphite	*R*_int_ = 0.101
Detector resolution: 13.6612 pixels mm^-1^	θ_max_ = 25.0°, θ_min_ = 1.8°
Thin–slice ω scans	*h* = −21→19
Absorption correction: multi-scan (*CrystalClear*; Rigaku, 2005)	*k* = 0→18
*T*_min_ = 0.221, *T*_max_ = 0.320	*l* = 0→20
7814 measured reflections	

### Refinement

**Table:**

Refinement on *F*^2^	Primary atom site location: structure-invariant direct methods
Least-squares matrix: full	Secondary atom site location: difference Fourier map
*R*[*F*^2^ > 2σ(*F*^2^)] = 0.048	Hydrogen site location: inferred from neighbouring sites
*wR*(*F*^2^) = 0.099	H atoms treated by a mixture of independent and constrained refinement
*S* = 0.87	*w* = 1/[σ^2^(*F*_o_^2^) + (0.0269*P*)^2^] where *P* = (*F*_o_^2^ + 2*F*_c_^2^)/3
7814 reflections	(Δ/σ)_max_ = 0.001
487 parameters	Δρ_max_ = 0.67 e Å^−^^3^
12 restraints	Δρ_min_ = −0.82 e Å^−^^3^

### Special details

**Table:**

Geometry. All e.s.d.'s (except the e.s.d. in the dihedral angle between two l.s. planes) are estimated using the full covariance matrix. The cell e.s.d.'s are taken into account individually in the estimation of e.s.d.'s in distances, angles and torsion angles; correlations between e.s.d.'s in cell parameters are only used when they are defined by crystal symmetry. An approximate (isotropic) treatment of cell e.s.d.'s is used for estimating e.s.d.'s involving l.s. planes.
Refinement. Refinement of *F*^2^ against ALL reflections. The weighted *R*-factor *wR* and goodness of fit *S* are based on *F*^2^, conventional *R*-factors *R* are based on *F*, with *F* set to zero for negative *F*^2^. The threshold expression of *F*^2^ > σ(*F*^2^) is used only for calculating *R*-factors(gt) *etc*. and is not relevant to the choice of reflections for refinement. *R*-factors based on *F*^2^ are statistically about twice as large as those based on *F*, and *R*- factors based on ALL data will be even larger.

### Fractional atomic coordinates and isotropic or equivalent isotropic displacement parameters (Å^2^)

**Table:**

	*x*	*y*	*z*	*U*_iso_*/*U*_eq_	
Br1	0.68214 (4)	0.55475 (5)	0.65807 (5)	0.0576 (2)	
Br2	0.56344 (4)	0.42590 (5)	0.83988 (5)	0.0558 (2)	
Br3	0.10334 (5)	0.61103 (5)	0.85539 (5)	0.0623 (3)	
Br4	0.14785 (5)	0.40766 (5)	0.65419 (5)	0.0564 (2)	
Cu1	0.85587 (5)	0.50884 (4)	0.73900 (5)	0.0322 (2)	
Cu2	0.39711 (4)	0.48668 (4)	0.76047 (5)	0.0311 (2)	
O1W	0.6265 (4)	0.3601 (3)	0.6868 (4)	0.0777 (17)	
H1W1	0.638 (5)	0.408 (3)	0.675 (5)	0.117*	
H1W2	0.608 (5)	0.370 (5)	0.723 (4)	0.117*	
O2W	0.6219 (4)	0.6163 (3)	0.8101 (5)	0.097 (2)	
H2W1	0.627 (6)	0.578 (4)	0.843 (5)	0.145*	
H2W2	0.644 (6)	0.602 (6)	0.779 (5)	0.145*	
O3W	0.0601 (5)	0.4038 (4)	0.7992 (5)	0.0903 (19)	
H3W1	0.075 (6)	0.408 (5)	0.761 (4)	0.135*	
H3W2	0.058 (6)	0.451 (3)	0.817 (6)	0.135*	
O4W	0.1901 (4)	0.6135 (3)	0.7066 (4)	0.0819 (18)	
H4W1	0.165 (5)	0.609 (5)	0.737 (5)	0.123*	
H4W2	0.191 (6)	0.566 (3)	0.686 (5)	0.123*	
N1	0.8212 (3)	0.4034 (3)	0.7847 (3)	0.0315 (13)	
H1	0.7663	0.4045	0.7627	0.038*	
N2	0.8619 (3)	0.4314 (3)	0.6499 (3)	0.0300 (13)	
N3	0.9094 (3)	0.6081 (3)	0.7058 (3)	0.0307 (13)	
H3	0.9628	0.6033	0.7384	0.037*	
N4	0.8610 (3)	0.5833 (3)	0.8355 (3)	0.0290 (13)	
N5	0.3402 (3)	0.3978 (3)	0.8025 (3)	0.0316 (13)	
H5	0.2872	0.4026	0.7689	0.038*	
N6	0.3864 (3)	0.4004 (3)	0.6719 (3)	0.0305 (13)	
N7	0.4310 (3)	0.5837 (3)	0.7016 (3)	0.0297 (13)	
H7	0.4859	0.5822	0.7214	0.036*	
N8	0.3920 (3)	0.5763 (3)	0.8397 (3)	0.0283 (13)	
C1	0.8070 (4)	0.3185 (4)	0.9007 (4)	0.056 (2)	
H1A	0.7496	0.3190	0.8707	0.084*	
H1B	0.8186	0.3187	0.9597	0.084*	
H1C	0.8295	0.2676	0.8862	0.084*	
C2	0.8440 (4)	0.3981 (4)	0.8775 (4)	0.0353 (17)	
C3	0.8082 (4)	0.4773 (4)	0.9036 (4)	0.0356 (17)	
H3A	0.7506	0.4769	0.8714	0.043*	
H3B	0.8154	0.4696	0.9618	0.043*	
C4	0.8388 (4)	0.5647 (4)	0.8955 (4)	0.0360 (18)	
C5	0.8401 (4)	0.6287 (4)	0.9606 (4)	0.056 (2)	
H5A	0.8697	0.6789	0.9563	0.083*	
H5B	0.8657	0.6035	1.0151	0.083*	
H5C	0.7859	0.6449	0.9525	0.083*	
C6	0.9357 (4)	0.3966 (4)	0.9225 (4)	0.050 (2)	
H6A	0.9562	0.3418	0.9135	0.075*	
H6B	0.9493	0.4051	0.9813	0.075*	
H6C	0.9592	0.4417	0.9010	0.075*	
C7	0.9612 (4)	0.6862 (4)	0.6111 (4)	0.049 (2)	
H7A	0.9416	0.7402	0.6230	0.074*	
H7B	0.9611	0.6875	0.5554	0.074*	
H7C	1.0152	0.6767	0.6504	0.074*	
C8	0.9063 (4)	0.6128 (4)	0.6181 (4)	0.0257 (15)	
C9	0.9413 (4)	0.5281 (3)	0.6002 (4)	0.0354 (17)	
H9A	0.9440	0.5334	0.5453	0.042*	
H9B	0.9965	0.5239	0.6402	0.042*	
C10	0.9008 (4)	0.4439 (4)	0.6020 (4)	0.0301 (16)	
C11	0.9109 (4)	0.3765 (4)	0.5437 (4)	0.056 (2)	
H11A	0.8869	0.3233	0.5513	0.083*	
H11B	0.9673	0.3676	0.5558	0.083*	
H11C	0.8848	0.3955	0.4869	0.083*	
C12	0.8204 (3)	0.6267 (4)	0.5564 (4)	0.0390 (17)	
H12A	0.7851	0.5864	0.5678	0.059*	
H12B	0.8182	0.6180	0.5005	0.059*	
H12C	0.8037	0.6844	0.5619	0.059*	
C13	0.8442 (4)	0.3282 (4)	0.7449 (4)	0.0408 (18)	
H13A	0.8147	0.2774	0.7499	0.049*	
H13B	0.9015	0.3166	0.7725	0.049*	
C14	0.8244 (4)	0.3485 (4)	0.6540 (4)	0.0407 (18)	
H14A	0.8453	0.3038	0.6283	0.049*	
H14B	0.7664	0.3519	0.6244	0.049*	
C15	0.8811 (4)	0.6858 (4)	0.7374 (4)	0.0436 (19)	
H15A	0.8249	0.6970	0.7033	0.052*	
H15B	0.9125	0.7357	0.7341	0.052*	
C16	0.8905 (4)	0.6707 (4)	0.8273 (4)	0.0445 (19)	
H16A	0.9468	0.6761	0.8637	0.053*	
H16B	0.8598	0.7135	0.8439	0.053*	
C17	0.2880 (4)	0.3393 (4)	0.9064 (4)	0.047 (2)	
H17A	0.2335	0.3470	0.8675	0.071*	
H17B	0.2898	0.3457	0.9624	0.071*	
H17C	0.3064	0.2827	0.8997	0.071*	
C18	0.3426 (4)	0.4072 (4)	0.8898 (4)	0.0311 (17)	
C19	0.3091 (4)	0.4961 (4)	0.8974 (4)	0.0382 (18)	
H19A	0.2539	0.4982	0.8570	0.046*	
H19B	0.3063	0.4992	0.9524	0.046*	
C20	0.3499 (4)	0.5763 (4)	0.8861 (4)	0.0319 (17)	
C21	0.3361 (4)	0.6534 (4)	0.9306 (4)	0.053 (2)	
H21A	0.3692	0.6494	0.9890	0.080*	
H21B	0.2803	0.6558	0.9237	0.080*	
H21C	0.3500	0.7046	0.9077	0.080*	
C22	0.4281 (4)	0.3979 (4)	0.9541 (4)	0.0451 (19)	
H22A	0.4460	0.3396	0.9542	0.068*	
H22B	0.4289	0.4122	1.0086	0.068*	
H22C	0.4634	0.4362	0.9402	0.068*	
C23	0.4381 (4)	0.6507 (4)	0.5737 (4)	0.058 (2)	
H23A	0.4141	0.7040	0.5806	0.087*	
H23B	0.4264	0.6410	0.5154	0.087*	
H23C	0.4955	0.6534	0.6034	0.087*	
C24	0.4033 (4)	0.5764 (4)	0.6086 (4)	0.0347 (17)	
C25	0.4387 (4)	0.4924 (4)	0.5897 (4)	0.0379 (17)	
H25A	0.4289	0.4921	0.5305	0.045*	
H25B	0.4967	0.4945	0.6195	0.045*	
C26	0.4094 (4)	0.4076 (4)	0.6102 (4)	0.0332 (17)	
C27	0.4104 (4)	0.3350 (4)	0.5538 (4)	0.050 (2)	
H27A	0.4651	0.3170	0.5662	0.076*	
H27B	0.3869	0.3537	0.4967	0.076*	
H27C	0.3798	0.2875	0.5622	0.076*	
C28	0.3121 (4)	0.5763 (4)	0.5687 (4)	0.048 (2)	
H28A	0.2908	0.5376	0.5987	0.072*	
H28B	0.2955	0.5579	0.5116	0.072*	
H28C	0.2923	0.6335	0.5705	0.072*	
C29	0.3663 (4)	0.3148 (4)	0.7806 (4)	0.048 (2)	
H29A	0.3339	0.2686	0.7897	0.058*	
H29B	0.4224	0.3043	0.8156	0.058*	
C30	0.3563 (4)	0.3175 (4)	0.6898 (4)	0.0461 (19)	
H30A	0.3861	0.2705	0.6779	0.055*	
H30B	0.2997	0.3108	0.6545	0.055*	
C31	0.4096 (4)	0.6656 (4)	0.7330 (4)	0.0411 (18)	
H31A	0.3523	0.6771	0.7053	0.049*	
H31B	0.4393	0.7133	0.7219	0.049*	
C32	0.4314 (4)	0.6557 (3)	0.8265 (4)	0.0370 (17)	
H32A	0.4895	0.6513	0.8550	0.044*	
H32B	0.4128	0.7053	0.8484	0.044*	

### Atomic displacement parameters (Å^2^)

**Table:**

	*U*^11^	*U*^22^	*U*^33^	*U*^12^	*U*^13^	*U*^23^
Br1	0.0473 (5)	0.0711 (5)	0.0510 (5)	0.0010 (4)	0.0144 (4)	0.0231 (5)
Br2	0.0455 (5)	0.0689 (5)	0.0543 (6)	0.0040 (4)	0.0203 (4)	0.0207 (4)
Br3	0.0591 (6)	0.0778 (6)	0.0436 (5)	0.0077 (4)	0.0121 (4)	−0.0088 (5)
Br4	0.0582 (6)	0.0606 (5)	0.0447 (5)	0.0065 (4)	0.0130 (4)	−0.0054 (4)
Cu1	0.0531 (6)	0.0252 (4)	0.0226 (5)	−0.0017 (4)	0.0191 (5)	0.0000 (4)
Cu2	0.0488 (6)	0.0245 (4)	0.0220 (5)	−0.0025 (4)	0.0156 (4)	0.0000 (4)
O1W	0.097 (5)	0.052 (3)	0.093 (5)	−0.008 (3)	0.046 (4)	−0.014 (3)
O2W	0.110 (5)	0.047 (3)	0.163 (7)	0.010 (3)	0.084 (5)	0.009 (4)
O3W	0.104 (5)	0.091 (4)	0.089 (6)	−0.009 (4)	0.051 (4)	0.025 (4)
O4W	0.092 (5)	0.068 (4)	0.088 (5)	−0.006 (4)	0.036 (4)	0.020 (4)
N1	0.039 (4)	0.032 (3)	0.019 (3)	0.005 (2)	0.006 (3)	0.005 (3)
N2	0.044 (4)	0.022 (3)	0.025 (3)	−0.001 (2)	0.015 (3)	0.000 (3)
N3	0.044 (4)	0.023 (3)	0.025 (3)	0.004 (2)	0.013 (3)	0.003 (3)
N4	0.040 (4)	0.023 (3)	0.027 (3)	−0.002 (2)	0.016 (3)	−0.003 (3)
N5	0.039 (3)	0.030 (3)	0.020 (3)	0.000 (2)	0.005 (3)	0.001 (3)
N6	0.038 (4)	0.025 (3)	0.031 (3)	−0.001 (2)	0.015 (3)	0.004 (3)
N7	0.037 (3)	0.030 (3)	0.022 (3)	0.002 (2)	0.011 (3)	−0.001 (3)
N8	0.038 (4)	0.024 (3)	0.025 (3)	0.000 (2)	0.014 (3)	0.006 (3)
C1	0.089 (6)	0.047 (5)	0.039 (5)	0.003 (4)	0.034 (5)	0.010 (4)
C2	0.049 (5)	0.033 (4)	0.021 (4)	0.002 (3)	0.010 (4)	0.008 (3)
C3	0.038 (4)	0.047 (5)	0.024 (4)	0.003 (3)	0.013 (3)	0.001 (4)
C4	0.034 (4)	0.045 (5)	0.033 (5)	0.006 (3)	0.017 (4)	0.003 (4)
C5	0.076 (6)	0.059 (5)	0.044 (5)	−0.007 (4)	0.037 (4)	−0.017 (4)
C6	0.054 (5)	0.062 (5)	0.027 (4)	0.025 (4)	0.007 (4)	0.005 (4)
C7	0.067 (5)	0.042 (4)	0.045 (5)	−0.011 (4)	0.028 (4)	0.009 (4)
C8	0.035 (4)	0.034 (4)	0.014 (4)	0.000 (3)	0.016 (3)	0.004 (3)
C9	0.045 (5)	0.035 (4)	0.031 (4)	0.000 (3)	0.020 (4)	−0.005 (3)
C10	0.027 (4)	0.037 (4)	0.021 (4)	0.005 (3)	0.003 (3)	0.003 (3)
C11	0.066 (6)	0.062 (5)	0.048 (5)	−0.011 (4)	0.031 (4)	−0.024 (4)
C12	0.037 (4)	0.053 (4)	0.026 (4)	0.003 (3)	0.010 (4)	0.005 (4)
C13	0.067 (5)	0.022 (4)	0.042 (5)	−0.003 (3)	0.030 (4)	0.003 (4)
C14	0.066 (5)	0.029 (4)	0.030 (5)	−0.006 (3)	0.021 (4)	−0.003 (3)
C15	0.072 (6)	0.024 (4)	0.045 (5)	0.004 (3)	0.033 (4)	0.002 (4)
C16	0.068 (5)	0.040 (4)	0.035 (5)	−0.005 (4)	0.031 (4)	−0.011 (4)
C17	0.054 (5)	0.050 (5)	0.041 (5)	−0.003 (4)	0.022 (4)	0.007 (4)
C18	0.030 (4)	0.033 (4)	0.035 (5)	0.002 (3)	0.017 (4)	0.007 (3)
C19	0.048 (5)	0.048 (5)	0.027 (4)	0.004 (4)	0.023 (4)	0.003 (4)
C20	0.037 (5)	0.034 (4)	0.015 (4)	0.011 (3)	−0.001 (3)	−0.001 (3)
C21	0.061 (5)	0.055 (5)	0.051 (5)	0.005 (4)	0.029 (4)	−0.012 (4)
C22	0.042 (5)	0.055 (5)	0.030 (4)	0.007 (3)	0.004 (4)	0.008 (4)
C23	0.085 (6)	0.055 (5)	0.038 (5)	−0.007 (4)	0.028 (4)	0.019 (4)
C24	0.049 (5)	0.041 (4)	0.011 (4)	0.002 (3)	0.008 (4)	0.010 (3)
C25	0.043 (4)	0.047 (4)	0.024 (4)	0.001 (4)	0.014 (3)	0.004 (4)
C26	0.029 (4)	0.038 (4)	0.029 (4)	0.005 (3)	0.005 (4)	0.002 (4)
C27	0.070 (6)	0.048 (5)	0.030 (5)	0.004 (4)	0.015 (4)	−0.009 (4)
C28	0.056 (5)	0.051 (5)	0.021 (4)	0.005 (4)	−0.001 (4)	0.001 (4)
C29	0.090 (6)	0.023 (4)	0.048 (5)	−0.003 (4)	0.043 (5)	0.005 (4)
C30	0.071 (5)	0.032 (4)	0.046 (5)	−0.010 (4)	0.034 (4)	−0.008 (4)
C31	0.064 (5)	0.026 (4)	0.033 (5)	−0.004 (3)	0.018 (4)	0.000 (3)
C32	0.061 (5)	0.021 (4)	0.031 (4)	−0.002 (3)	0.020 (4)	−0.007 (3)

### Geometric parameters (Å, °)

**Table:**

Br1—Cu1	2.9734 (11)	C9—H9A	0.9700
Br2—Cu2	2.9229 (11)	C9—H9B	0.9700
Cu1—N2	1.986 (5)	C10—C11	1.509 (8)
Cu1—N4	2.000 (5)	C11—H11A	0.9600
Cu1—N3	2.007 (4)	C11—H11B	0.9600
Cu1—N1	2.011 (4)	C11—H11C	0.9600
Cu2—N8	1.977 (5)	C12—H12A	0.9600
Cu2—N6	1.986 (5)	C12—H12B	0.9600
Cu2—N5	2.004 (5)	C12—H12C	0.9600
Cu2—N7	2.032 (4)	C13—C14	1.506 (7)
O1W—H1W1	0.824 (19)	C13—H13A	0.9700
O1W—H1W2	0.83 (2)	C13—H13B	0.9700
O2W—H2W1	0.80 (2)	C14—H14A	0.9700
O2W—H2W2	0.81 (2)	C14—H14B	0.9700
O3W—H3W1	0.81 (2)	C15—C16	1.515 (8)
O3W—H3W2	0.80 (2)	C15—H15A	0.9700
O4W—H4W1	0.81 (2)	C15—H15B	0.9700
O4W—H4W2	0.813 (19)	C16—H16A	0.9700
N1—C13	1.488 (7)	C16—H16B	0.9700
N1—C2	1.499 (7)	C17—C18	1.535 (8)
N1—H1	0.9100	C17—H17A	0.9600
N2—C10	1.279 (7)	C17—H17B	0.9600
N2—C14	1.464 (7)	C17—H17C	0.9600
N3—C15	1.488 (7)	C18—C22	1.522 (8)
N3—C8	1.496 (7)	C18—C19	1.529 (8)
N3—H3	0.9108	C19—C20	1.491 (8)
N4—C4	1.273 (7)	C19—H19A	0.9700
N4—C16	1.479 (7)	C19—H19B	0.9700
N5—C29	1.465 (7)	C20—C21	1.491 (8)
N5—C18	1.498 (7)	C21—H21A	0.9600
N5—H5	0.9089	C21—H21B	0.9600
N6—C26	1.282 (7)	C21—H21C	0.9600
N6—C30	1.471 (7)	C22—H22A	0.9600
N7—C31	1.486 (7)	C22—H22B	0.9600
N7—C24	1.494 (7)	C22—H22C	0.9600
N7—H7	0.9101	C23—C24	1.535 (8)
N8—C20	1.287 (7)	C23—H23A	0.9600
N8—C32	1.479 (7)	C23—H23B	0.9600
C1—C2	1.523 (8)	C23—H23C	0.9600
C1—H1A	0.9600	C24—C28	1.513 (8)
C1—H1B	0.9600	C24—C25	1.536 (8)
C1—H1C	0.9600	C25—C26	1.506 (8)
C2—C6	1.528 (8)	C25—H25A	0.9700
C2—C3	1.528 (8)	C25—H25B	0.9700
C3—C4	1.488 (8)	C26—C27	1.493 (8)
C3—H3A	0.9700	C27—H27A	0.9600
C3—H3B	0.9700	C27—H27B	0.9600
C4—C5	1.493 (8)	C27—H27C	0.9600
C5—H5A	0.9600	C28—H28A	0.9600
C5—H5B	0.9600	C28—H28B	0.9600
C5—H5C	0.9600	C28—H28C	0.9600
C6—H6A	0.9600	C29—C30	1.509 (8)
C6—H6B	0.9600	C29—H29A	0.9700
C6—H6C	0.9600	C29—H29B	0.9700
C7—C8	1.538 (7)	C30—H30A	0.9700
C7—H7A	0.9600	C30—H30B	0.9700
C7—H7B	0.9600	C31—C32	1.518 (8)
C7—H7C	0.9600	C31—H31A	0.9700
C8—C12	1.518 (7)	C31—H31B	0.9700
C8—C9	1.536 (7)	C32—H32A	0.9700
C9—C10	1.499 (8)	C32—H32B	0.9700
			
N2—Cu1—N4	174.3 (2)	C8—C12—H12A	109.5
N2—Cu1—N3	94.19 (19)	C8—C12—H12B	109.5
N4—Cu1—N3	85.20 (19)	H12A—C12—H12B	109.5
N2—Cu1—N1	85.73 (19)	C8—C12—H12C	109.5
N4—Cu1—N1	93.92 (19)	H12A—C12—H12C	109.5
N3—Cu1—N1	170.3 (2)	H12B—C12—H12C	109.5
N2—Cu1—Br1	97.60 (14)	N1—C13—C14	108.6 (5)
N4—Cu1—Br1	88.02 (14)	N1—C13—H13A	110.0
N3—Cu1—Br1	102.03 (14)	C14—C13—H13A	110.0
N1—Cu1—Br1	87.58 (14)	N1—C13—H13B	110.0
N8—Cu2—N6	172.1 (2)	C14—C13—H13B	110.0
N8—Cu2—N5	94.13 (19)	H13A—C13—H13B	108.4
N6—Cu2—N5	84.2 (2)	N2—C14—C13	107.5 (5)
N8—Cu2—N7	85.69 (19)	N2—C14—H14A	110.2
N6—Cu2—N7	94.32 (19)	C13—C14—H14A	110.2
N5—Cu2—N7	168.0 (2)	N2—C14—H14B	110.2
N8—Cu2—Br2	101.90 (14)	C13—C14—H14B	110.2
N6—Cu2—Br2	86.00 (14)	H14A—C14—H14B	108.5
N5—Cu2—Br2	99.99 (14)	N3—C15—C16	109.1 (5)
N7—Cu2—Br2	91.81 (14)	N3—C15—H15A	109.9
H1W1—O1W—H1W2	103 (8)	C16—C15—H15A	109.9
H2W1—O2W—H2W2	108 (10)	N3—C15—H15B	109.9
H3W1—O3W—H3W2	109 (9)	C16—C15—H15B	109.9
H4W1—O4W—H4W2	108 (9)	H15A—C15—H15B	108.3
C13—N1—C2	116.0 (5)	N4—C16—C15	109.5 (5)
C13—N1—Cu1	106.1 (3)	N4—C16—H16A	109.8
C2—N1—Cu1	117.6 (4)	C15—C16—H16A	109.8
C13—N1—H1	105.3	N4—C16—H16B	109.8
C2—N1—H1	105.3	C15—C16—H16B	109.8
Cu1—N1—H1	105.3	H16A—C16—H16B	108.2
C10—N2—C14	122.3 (5)	C18—C17—H17A	109.5
C10—N2—Cu1	127.5 (4)	C18—C17—H17B	109.5
C14—N2—Cu1	109.6 (4)	H17A—C17—H17B	109.5
C15—N3—C8	116.3 (4)	C18—C17—H17C	109.5
C15—N3—Cu1	104.6 (4)	H17A—C17—H17C	109.5
C8—N3—Cu1	119.0 (3)	H17B—C17—H17C	109.5
C15—N3—H3	105.2	N5—C18—C22	111.4 (5)
C8—N3—H3	105.2	N5—C18—C19	108.1 (5)
Cu1—N3—H3	105.2	C22—C18—C19	110.0 (5)
C4—N4—C16	121.1 (5)	N5—C18—C17	109.9 (5)
C4—N4—Cu1	127.9 (4)	C22—C18—C17	109.5 (5)
C16—N4—Cu1	110.9 (4)	C19—C18—C17	107.8 (5)
C29—N5—C18	117.0 (5)	C20—C19—C18	120.9 (5)
C29—N5—Cu2	104.9 (4)	C20—C19—H19A	107.1
C18—N5—Cu2	118.0 (4)	C18—C19—H19A	107.1
C29—N5—H5	105.3	C20—C19—H19B	107.1
C18—N5—H5	105.2	C18—C19—H19B	107.1
Cu2—N5—H5	105.2	H19A—C19—H19B	106.8
C26—N6—C30	120.5 (5)	N8—C20—C21	124.6 (6)
C26—N6—Cu2	127.7 (4)	N8—C20—C19	120.7 (5)
C30—N6—Cu2	111.4 (4)	C21—C20—C19	114.7 (6)
C31—N7—C24	115.5 (5)	C20—C21—H21A	109.5
C31—N7—Cu2	106.5 (3)	C20—C21—H21B	109.5
C24—N7—Cu2	116.1 (4)	H21A—C21—H21B	109.5
C31—N7—H7	105.9	C20—C21—H21C	109.5
C24—N7—H7	106.0	H21A—C21—H21C	109.5
Cu2—N7—H7	106.0	H21B—C21—H21C	109.5
C20—N8—C32	121.4 (5)	C18—C22—H22A	109.5
C20—N8—Cu2	128.3 (4)	C18—C22—H22B	109.5
C32—N8—Cu2	109.2 (4)	H22A—C22—H22B	109.5
C2—C1—H1A	109.5	C18—C22—H22C	109.5
C2—C1—H1B	109.5	H22A—C22—H22C	109.5
H1A—C1—H1B	109.5	H22B—C22—H22C	109.5
C2—C1—H1C	109.5	C24—C23—H23A	109.5
H1A—C1—H1C	109.5	C24—C23—H23B	109.5
H1B—C1—H1C	109.5	H23A—C23—H23B	109.5
N1—C2—C1	110.8 (5)	C24—C23—H23C	109.5
N1—C2—C6	110.6 (5)	H23A—C23—H23C	109.5
C1—C2—C6	110.0 (5)	H23B—C23—H23C	109.5
N1—C2—C3	107.2 (5)	N7—C24—C28	110.7 (5)
C1—C2—C3	107.7 (5)	N7—C24—C23	110.1 (5)
C6—C2—C3	110.5 (5)	C28—C24—C23	110.3 (5)
C4—C3—C2	119.7 (5)	N7—C24—C25	107.2 (5)
C4—C3—H3A	107.4	C28—C24—C25	111.2 (5)
C2—C3—H3A	107.4	C23—C24—C25	107.1 (5)
C4—C3—H3B	107.4	C26—C25—C24	119.0 (5)
C2—C3—H3B	107.4	C26—C25—H25A	107.6
H3A—C3—H3B	106.9	C24—C25—H25A	107.6
N4—C4—C3	121.4 (6)	C26—C25—H25B	107.6
N4—C4—C5	122.6 (6)	C24—C25—H25B	107.6
C3—C4—C5	116.0 (6)	H25A—C25—H25B	107.0
C4—C5—H5A	109.5	N6—C26—C27	124.2 (6)
C4—C5—H5B	109.5	N6—C26—C25	121.2 (6)
H5A—C5—H5B	109.5	C27—C26—C25	114.6 (6)
C4—C5—H5C	109.5	C26—C27—H27A	109.5
H5A—C5—H5C	109.5	C26—C27—H27B	109.5
H5B—C5—H5C	109.5	H27A—C27—H27B	109.5
C2—C6—H6A	109.5	C26—C27—H27C	109.5
C2—C6—H6B	109.5	H27A—C27—H27C	109.5
H6A—C6—H6B	109.5	H27B—C27—H27C	109.5
C2—C6—H6C	109.5	C24—C28—H28A	109.5
H6A—C6—H6C	109.5	C24—C28—H28B	109.5
H6B—C6—H6C	109.5	H28A—C28—H28B	109.5
C8—C7—H7A	109.5	C24—C28—H28C	109.5
C8—C7—H7B	109.5	H28A—C28—H28C	109.5
H7A—C7—H7B	109.5	H28B—C28—H28C	109.5
C8—C7—H7C	109.5	N5—C29—C30	108.5 (5)
H7A—C7—H7C	109.5	N5—C29—H29A	110.0
H7B—C7—H7C	109.5	C30—C29—H29A	110.0
N3—C8—C12	111.1 (5)	N5—C29—H29B	110.0
N3—C8—C9	107.4 (5)	C30—C29—H29B	110.0
C12—C8—C9	111.2 (5)	H29A—C29—H29B	108.4
N3—C8—C7	109.4 (5)	N6—C30—C29	109.4 (5)
C12—C8—C7	110.3 (5)	N6—C30—H30A	109.8
C9—C8—C7	107.3 (5)	C29—C30—H30A	109.8
C10—C9—C8	120.2 (5)	N6—C30—H30B	109.8
C10—C9—H9A	107.3	C29—C30—H30B	109.8
C8—C9—H9A	107.3	H30A—C30—H30B	108.2
C10—C9—H9B	107.3	N7—C31—C32	107.6 (5)
C8—C9—H9B	107.3	N7—C31—H31A	110.2
H9A—C9—H9B	106.9	C32—C31—H31A	110.2
N2—C10—C9	122.0 (6)	N7—C31—H31B	110.2
N2—C10—C11	123.7 (6)	C32—C31—H31B	110.2
C9—C10—C11	114.2 (6)	H31A—C31—H31B	108.5
C10—C11—H11A	109.5	N8—C32—C31	107.6 (5)
C10—C11—H11B	109.5	N8—C32—H32A	110.2
H11A—C11—H11B	109.5	C31—C32—H32A	110.2
C10—C11—H11C	109.5	N8—C32—H32B	110.2
H11A—C11—H11C	109.5	C31—C32—H32B	110.2
H11B—C11—H11C	109.5	H32A—C32—H32B	108.5
			
N2—Cu1—N1—C13	17.1 (4)	C2—C3—C4—C5	−146.2 (6)
N4—Cu1—N1—C13	−157.3 (4)	C15—N3—C8—C12	−60.5 (6)
Br1—Cu1—N1—C13	114.9 (4)	Cu1—N3—C8—C12	66.1 (5)
N2—Cu1—N1—C2	148.9 (4)	C15—N3—C8—C9	177.7 (5)
N4—Cu1—N1—C2	−25.5 (4)	Cu1—N3—C8—C9	−55.8 (6)
Br1—Cu1—N1—C2	−113.3 (4)	C15—N3—C8—C7	61.5 (7)
N3—Cu1—N2—C10	10.1 (6)	Cu1—N3—C8—C7	−171.9 (4)
N1—Cu1—N2—C10	−160.2 (6)	N3—C8—C9—C10	62.6 (7)
Br1—Cu1—N2—C10	112.8 (5)	C12—C8—C9—C10	−59.1 (7)
N3—Cu1—N2—C14	−178.6 (4)	C7—C8—C9—C10	−179.8 (5)
N1—Cu1—N2—C14	11.1 (4)	C14—N2—C10—C9	−177.9 (5)
Br1—Cu1—N2—C14	−75.9 (4)	Cu1—N2—C10—C9	−7.7 (9)
N2—Cu1—N3—C15	155.8 (4)	C14—N2—C10—C11	1.3 (10)
N4—Cu1—N3—C15	−29.9 (4)	Cu1—N2—C10—C11	171.6 (4)
Br1—Cu1—N3—C15	57.1 (4)	C8—C9—C10—N2	−30.9 (9)
N2—Cu1—N3—C8	23.9 (4)	C8—C9—C10—C11	149.8 (6)
N4—Cu1—N3—C8	−161.8 (4)	C2—N1—C13—C14	−174.3 (5)
Br1—Cu1—N3—C8	−74.8 (4)	Cu1—N1—C13—C14	−41.6 (6)
N3—Cu1—N4—C4	−177.4 (6)	C10—N2—C14—C13	135.2 (6)
N1—Cu1—N4—C4	−7.1 (6)	Cu1—N2—C14—C13	−36.6 (6)
Br1—Cu1—N4—C4	80.3 (6)	N1—C13—C14—N2	52.3 (7)
N3—Cu1—N4—C16	6.4 (4)	C8—N3—C15—C16	−178.6 (5)
N1—Cu1—N4—C16	176.7 (4)	Cu1—N3—C15—C16	47.9 (6)
Br1—Cu1—N4—C16	−95.8 (4)	C4—N4—C16—C15	−157.7 (6)
N8—Cu2—N5—C29	−155.8 (4)	Cu1—N4—C16—C15	18.7 (6)
N6—Cu2—N5—C29	31.9 (4)	N3—C15—C16—N4	−44.7 (7)
N7—Cu2—N5—C29	115.4 (9)	C29—N5—C18—C22	61.9 (7)
Br2—Cu2—N5—C29	−53.0 (4)	Cu2—N5—C18—C22	−64.8 (6)
N8—Cu2—N5—C18	−23.5 (4)	C29—N5—C18—C19	−177.1 (5)
N6—Cu2—N5—C18	164.2 (4)	Cu2—N5—C18—C19	56.2 (6)
N7—Cu2—N5—C18	−112.2 (9)	C29—N5—C18—C17	−59.7 (7)
Br2—Cu2—N5—C18	79.4 (4)	Cu2—N5—C18—C17	173.6 (4)
N5—Cu2—N6—C26	178.8 (6)	N5—C18—C19—C20	−62.3 (7)
N7—Cu2—N6—C26	10.8 (6)	C22—C18—C19—C20	59.6 (7)
Br2—Cu2—N6—C26	−80.7 (5)	C17—C18—C19—C20	178.9 (5)
N5—Cu2—N6—C30	−8.4 (4)	C32—N8—C20—C21	0.1 (9)
N7—Cu2—N6—C30	−176.4 (4)	Cu2—N8—C20—C21	−166.6 (4)
Br2—Cu2—N6—C30	92.1 (4)	C32—N8—C20—C19	178.7 (5)
N8—Cu2—N7—C31	−16.3 (4)	Cu2—N8—C20—C19	12.0 (9)
N6—Cu2—N7—C31	155.8 (4)	C18—C19—C20—N8	27.5 (9)
N5—Cu2—N7—C31	73.3 (10)	C18—C19—C20—C21	−153.8 (6)
Br2—Cu2—N7—C31	−118.1 (4)	C31—N7—C24—C28	−64.3 (7)
N8—Cu2—N7—C24	−146.5 (4)	Cu2—N7—C24—C28	61.5 (6)
N6—Cu2—N7—C24	25.6 (4)	C31—N7—C24—C23	58.0 (7)
N5—Cu2—N7—C24	−56.9 (11)	Cu2—N7—C24—C23	−176.2 (4)
Br2—Cu2—N7—C24	111.7 (4)	C31—N7—C24—C25	174.3 (5)
N5—Cu2—N8—C20	−12.6 (5)	Cu2—N7—C24—C25	−60.0 (6)
N7—Cu2—N8—C20	155.3 (5)	N7—C24—C25—C26	67.1 (7)
Br2—Cu2—N8—C20	−113.7 (5)	C28—C24—C25—C26	−54.1 (7)
N5—Cu2—N8—C32	179.4 (4)	C23—C24—C25—C26	−174.7 (5)
N7—Cu2—N8—C32	−12.6 (4)	C30—N6—C26—C27	−1.8 (9)
Br2—Cu2—N8—C32	78.3 (4)	Cu2—N6—C26—C27	170.3 (4)
C13—N1—C2—C1	−57.8 (7)	C30—N6—C26—C25	178.6 (5)
Cu1—N1—C2—C1	175.1 (4)	Cu2—N6—C26—C25	−9.2 (9)
C13—N1—C2—C6	64.5 (6)	C24—C25—C26—N6	−31.2 (9)
Cu1—N1—C2—C6	−62.7 (6)	C24—C25—C26—C27	149.2 (6)
C13—N1—C2—C3	−175.0 (5)	C18—N5—C29—C30	177.8 (5)
Cu1—N1—C2—C3	57.9 (6)	Cu2—N5—C29—C30	−49.3 (6)
N1—C2—C3—C4	−66.2 (7)	C26—N6—C30—C29	156.6 (6)
C1—C2—C3—C4	174.6 (6)	Cu2—N6—C30—C29	−16.8 (6)
C6—C2—C3—C4	54.5 (8)	N5—C29—C30—N6	44.1 (7)
C16—N4—C4—C3	179.4 (5)	C24—N7—C31—C32	171.8 (5)
Cu1—N4—C4—C3	3.6 (9)	Cu2—N7—C31—C32	41.3 (5)
C16—N4—C4—C5	0.5 (10)	C20—N8—C32—C31	−130.3 (6)
Cu1—N4—C4—C5	−175.3 (4)	Cu2—N8—C32—C31	38.6 (6)
C2—C3—C4—N4	34.9 (9)	N7—C31—C32—N8	−53.3 (6)

### Hydrogen-bond geometry (Å, °)

**Table:**

*D*—H···*A*	*D*—H	H···*A*	*D*···*A*	*D*—H···*A*
O1W—H1W1···Br1	0.82 (2)	2.46 (2)	3.274 (5)	173 (8)
O1W—H1W2···Br2	0.83 (2)	2.58 (3)	3.395 (6)	170 (7)
O2W—H2W1···Br2	0.80 (2)	2.61 (8)	3.238 (6)	136 (9)
O2W—H2W2···Br1	0.81 (2)	2.53 (3)	3.328 (7)	168 (10)
O3W—H3W1···Br4	0.81 (2)	2.62 (3)	3.417 (6)	169 (10)
O3W—H3W2···Br3	0.80 (2)	2.61 (4)	3.362 (6)	156 (8)
O4W—H4W1···Br3	0.81 (2)	2.66 (2)	3.461 (6)	170 (8)
O4W—H4W2···Br4	0.81 (2)	2.58 (4)	3.327 (5)	155 (7)
N1—H1···O1W	0.91	2.44	3.317 (8)	161.
N1—H1···Br1	0.91	2.99	3.519 (5)	119.
N3—H3···Br3^i^	0.91	2.57	3.457 (5)	166.
N5—H5···Br4	0.91	2.53	3.432 (5)	170.
N7—H7···O2W	0.91	2.40	3.257 (9)	158.
N7—H7···Br2	0.91	3.14	3.612 (5)	115.

Symmetry codes: (i) *x*+1, *y*, *z*.
